# Kefir Consumption and Health Effects Based on Human Clinical Trials: An Overview of Literature

**DOI:** 10.3390/healthcare14050652

**Published:** 2026-03-04

**Authors:** Sabina Fijan, Petra Povalej Bržan, Maja Šikić Pogačar, Petra Klanjšek

**Affiliations:** 1 Faculty of Health Sciences, University of Maribor, Žitna Ulica 15, 2000 Maribor, Slovenia; petra.klanjsek@um.si; 2 Faculty of Medicine, University of Maribor, Taborska Ulica 8, 2000 Maribor, Slovenia; petra.povalej@um.si (P.P.B.); maja.sikic@um.si (M.Š.P.); 3 Faculty of Electrical Engineering and Computer Science, University of Maribor, Koroška Cesta 46, 2000 Maribor, Slovenia

**Keywords:** kefir, health benefits, randomized controlled trials, beneficial microbes

## Abstract

Kefir is a traditional fermented milk beverage characterized by a complex community of lactic acid bacteria, acetic acid bacteria and yeasts that contributes to its unique sensory and nutritional properties. Regular consumption of kefir has been associated with a wide range of potential health benefits. This review aimed to evaluate the available clinical evidence on kefir consumption and its impact on human health. A literature search of the databases PubMed, Web of Science, and Scopus was conducted up to 30 August 2025. Eligible studies were human clinical trials investigating kefir as a fermented milk beverage without the addition of defined probiotic strains, prebiotics, or synbiotics. A total of 28 clinical studies were identified and included diverse study designs, such as crossover trials, parallel-group randomized controlled trials, multi-arm trials, early-phase exploratory studies, and pilot studies. Kefir intake showed potential benefits for gut microbiota modulation, metabolic parameters, inflammatory markers, immune function, and gastrointestinal health. However, interpretation of these findings is limited due to substantial heterogeneity in kefir preparation, microbial composition, dosage, intervention duration, study populations, and outcome measures. Consequently, although kefir may offer multiple health benefits, the overall strength of evidence remains limited. Larger, well-designed clinical trials with standardized kefir interventions are needed to better define kefir’s efficacy in specific populations and health conditions.

## 1. Introduction

Kefir is a traditional fermented milk beverage that originates from the Caucasus region and Eastern Europe, and was used in ancient times. The origins of kefir predate written records and has been consumed for centuries as part of the daily diet and valued for its perceived health-promoting properties. Owing to its long history of consumption, kefir has attracted increasing scientific interest as a functional food, prompting clinical investigations into its potential health effects [1,2].

Kefir is traditionally produced by fermenting milk with kefir grains, which consist of a complex microbial community of lactic acid bacteria (including *Lactobacillus kefiranofaciens*, *Lactobacillus helveticus*, *Lentilactobacillus kefiri*, *Lentilactobacillus parakefiri*, *Limosilactobacillus fermentum*, *Levilactobacillus brevis*, *Lacticaseibacillus paracasei*, *Lactiplantibacillus plantarum*, *Lactococcus lactis*), acetic acid bacteria (including *Acetobacter* spp.), and yeasts (including *Kluyveromyces marxianus*, *Kluyveromyces lactis* var. *lactis*, *Debaryomyces hansenii*, *Dekkera anomala*, *Saccharomyces cerevisiae*, *Torulaspora delbrueckii*, *Pichia fermentans*) [3,4,5,6,7,8].

The relative abundance and species composition of these microorganisms vary depending on the geographic origin of the grains, fermentation conditions, and substrate. Within the kefir grains, microorganisms are embedded in a polysaccharide matrix known as kefiran, which supports microbial stability and symbiosis. Through their metabolic activity, kefir microorganisms produce a wide array of bioactive metabolites, including organic acids (such as lactic and acetic acids), ethanol, carbon dioxide, bioactive peptides, exopolysaccharides, vitamins, and volatile compounds, which collectively contribute to kefir’s sensory properties and its potential health effects [3,4,5,6]. This unique microbial composition gives kefir a distinctive microbial richness and metabolic activity that differentiate it from other fermented dairy products. The fermentation process not only enhances the beverage’s digestibility and nutrient bioavailability but also generates a variety of bioactive compounds and peptides with potential health-promoting properties [6,9]. In addition to traditional grain-fermented kefir, commercially available kefir products are often produced using defined starter cultures designed to replicate the microbial profile of kefir grains, although they may differ in microbial diversity and metabolite production [7,10,11,12]. The term “water kefir” is used for a beverage produced with water kefir grains in a sugar-based solution. However, milk kefir and water kefir are distinct fermentation systems, characterized by different microbial consortia, substrates, and metabolite profiles [13,14]. Due to these fundamental differences, they should not be considered interchangeable when interpreting health-related findings.

According to the International Scientific Association for Probiotics and Prebiotics (ISAPP), kefir—as a fermented milk beverage—does not meet the definition of a probiotic [15,16]. Marco et al. [16] emphasize that fermented foods and probiotics are related but distinct categories. Fermented foods are defined as foods made through desired microbial growth and enzymatic transformations, and their primary purpose is to improve shelf life, safety, sensory properties, or nutritional value. Importantly, while these foods contain live microorganisms at the time of consumption, the presence of live microbes alone is not sufficient to qualify them as probiotics. To meet the definition of a probiotic, microorganisms must be well-defined at the strain level, demonstrate proven health benefits in controlled clinical studies, and be present in the product at an effective dose at the time of consumption [15,17].

Fermented foods generally do not meet these criteria because their microbial composition is highly variable, often not characterized at the strain level, and typically not standardized across batches or producers. In addition, most fermented foods have not undergone strain-specific clinical trials required to substantiate a probiotic health claim. For these reasons, Marco et al. [16] clearly distinguish fermented foods from probiotics, even though some microbes present in fermented foods may individually qualify as probiotics when isolated, identified, and clinically validated. However, some microbial strains isolated from kefir, such as *Lentilactobacillus kefiri* LKF01, *Lactococcus lactis* subsp. *cremoris* YRC3780, *Bifidobacterium longum* BL986, *Limosilactobacillus fermentum* LF26, *Lactobacillus helveticus* LH43, *Streptococcus thermophilus* ST30 and others, have shown health benefits in clinical studies [18,19,20,21,22]. While these studies showed beneficial effects, these benefits cannot be extrapolated to all kefir beverages, since the microbial composition varies considerably among products, the concentrations of specific microorganisms differ widely, and not all kefir preparations contain the specific strains that are effective.

Regular consumption of kefir has been associated with a wide spectrum of benefits, including improved gut health, modulation of intestinal microbiota, enhanced immune function, and protective effects against metabolic, cardiovascular, and gastrointestinal disorders [23,24]. Kefir also exhibits antimicrobial, anti-inflammatory, antioxidant, and anticarcinogenic activities, and is therefore an important part of nutritional science and functional food research [25,26]. While many of these effects have been demonstrated in animal and in vitro studies, clinical studies continue to explore kefir’s role in human health and disease prevention [27,28,29]. Kefir is also associated with high acceptance, particularly regarding its health benefits [30,31].

While prior reviews have summarized evidence from randomized controlled trials, most have focused on general outcomes in healthy adults and specific populations such as athletes, patients with metabolic syndrome, inflammatory bowel disease, osteoporosis, fibromyalgia, functional constipation, and periodontal disease [27,28,29,32,33,34]. These reviews report limited and inconsistent efficacy data, often highlighting a high risk of bias, small sample sizes and a lack of population-specific analyses. Importantly, meaningful conclusions are further impaired by substantial heterogeneity in kefir interventions, including differences in preparation methods, microbial composition, and—critically—dosage and duration of consumption. The reviewed studies included a wide range of kefir products, from traditional kefir, produced using kefir grains (known as pitcher-fermented kefir), to commercially manufactured or probiotic-enriched formulations in which defined probiotic strains were added before or after fermentation. These products were administered in widely varying amounts, making direct comparison challenging. Outcomes assessed also vary considerably, from gut microbiota composition and inflammatory markers to metabolic, cognitive, and appetite-related parameters. Several recently published randomized controlled trials have not yet been incorporated into these existing reviews. Although many in vitro and animal studies have reported beneficial effects of kefir, these findings cannot be directly extrapolated to humans. Since dietary recommendations and health-related conclusions should primarily rely on human data, this review focuses exclusively on clinical trials in humans to provide a more clinically meaningful evaluation of kefir consumption.

To address these gaps, the present review adopts a population-, intervention-, and outcome-specific approach, aiming to provide a more nuanced and critical synthesis of the evidence, while explicitly acknowledging the limitations imposed by the lack of standardized kefir dosing and intervention protocols.

## 2. Search Strategy and Study Selection

A literature review was performed to assess the health effects of milk-based kefir consumption. Electronic databases (PubMed, Web of Science, and Scopus) were searched using the search strategy listed in Table 1. Google Scholar was used as a bibliographic search engine to identify additional relevant studies. Further studies were identified by screening the reference lists of relevant articles.

Studies were included if they investigated commercial or industrially produced kefir as a fermented milk product obtained through microbial fermentation without added probiotic strains and additional lyophilisation, and reported human health outcomes that were published in peer-reviewed English-language journals.

Multi-arm trials were included when one study arm investigated kefir consumption as a fermented milk product, even if additional comparator arms included probiotic supplements or other interventions; only outcomes related to kefir consumption were considered in the qualitative synthesis.

## 3. Results

### 3.1. Overview of the Included Clinical Trials on Kefir Consumption

Based on the predefined inclusion criteria, a total of 28 studies evaluating the health effects of kefir consumption were identified [35,36,37,38,39,40,41,42,43,44,45,46,47,48,49,50,51,52,53,54,55,56,57,58,59,60,61,62]. Studies were conducted primarily in Turkey (13), Iran (4), and the United States (6), with individual studies from Slovenia, Brazil, Ireland, Taiwan, and Canada.

These studies included diverse populations, ranging from healthy adults to individuals with specific metabolic, gastrointestinal, or immunological conditions, and encompassed a variety of interventions in terms of kefir type, dosage, and duration.

Data extracted for each study included author(s), year, study design, population, kefir dosage and duration, outcomes, and main findings. The study designs (e.g., randomized controlled trials, double-blind, single-blind, and open-label studies) were named according to standard clinical research methodology [63,64]. Parallel-group randomized controlled trials allocate participants to either an intervention or a control group for the entire study period, allowing direct comparison between groups. In crossover trials, participants received both the intervention and the control in sequential periods separated by a washout phase, which reduces inter-individual variability but may be influenced by potential carryover effects. Open-label and single-blind designs carry a higher risk of expectation bias compared to double-blind trials. These methodological differences should be considered when interpreting and comparing outcomes across studies.

For clarity and ease of comparison, the studies are presented in Table 2 in descending chronological order and, within each year, alphabetically by the first author. Table 2 summarizes key study characteristics, including design, population, kefir intervention details, outcomes measured, and main findings, allowing readers to readily assess patterns, consistencies, and variability across the evidence base. Due to the distinctive sensory properties of kefir, true double-blinding was not feasible and is noted as a limitation of the studies.

The clinical studies investigating kefir consumption used diverse study designs. Eight studies were crossover trials [40,41,43,45,52,54,57,61,62], while eleven were two-arm parallel-group randomized controlled trials (RCTs) [35,37,42,44,46,47,56,58,59,60,66]. Several studies used multi-arm designs in which kefir was compared with probiotic supplements, unfermented dairy products, or standard care, allowing differentiation between the effects of kefir as a complex fermented milk beverage matrix and those of defined probiotic formulations. Six publications described three-arm parallel-group RCTs [39,48,49,50,51,57]. One four-arm study [53] and two single-arm studies [38,55] were also included. Four publications originated from two clinical trials [42,46,49,51].

These studies examined a broad spectrum of health outcomes. Metabolic parameters—such as lipid profile, glucose, insulin, blood pressure, body composition, metabolic syndrome, and NAFLD—were among the most frequently investigated parameters and showed that kefir intake was associated with improved metabolic and cardiovascular parameters, including HDL-C, LDL-C, ApoA1, triglycerides, glycemic index, blood pressure, fat-free mass, and reduced cardiovascular risk, with effects often dependent on microbial composition and specific strains [35,40,42,43,44,46,49,51,52,62]. Several studies focused on gut microbiota composition and gastrointestinal function, assessing microbial diversity, short-chain fatty acid production, constipation, diarrhea, antibiotic-associated diarrhea, IBD symptoms, and lactose digestion and found that kefir increased beneficial bacteria such as *Lactobacillus*, *Bifidobacterium*, *Akkermansia*, and *Faecalibacterium*, improved stool frequency and consistency, reduced constipation and antibiotic-associated diarrhea, and enhanced lactose tolerance [37,38,47,55,59,61]. Other investigations addressed immune and inflammatory markers and found that kefir consumption was associated with reductions in pro-inflammatory cytokines, CRP, TNF-α, and IL-6, improvements in periodontal health, and decreased side effects of *Helicobacter pylori* therapy [39,40,42,45,46,56,57,58,60]. Additional outcomes included cognitive and mood-related measures such as memory, mood, and stress regulation. Studies reported that kefir supplementation improved hippocampal-dependent memory and mood regulation [41,45]. Bone metabolism parameters (bone mineral density, serum calcium, parathyroid hormone, β-CTX) were also examined, and several investigations found beneficial effects on these markers [66]. Oral health parameters were also investigated, with kefir consumption leading to reduced *Streptococcus mutans* and *Lactobacillus* counts, and showing effects comparable to sodium fluoride rinses in tooth caries prevention [48,54,57]. Athletic performance was assessed, and it was found that kefir intake improved endurance, VO_2_ max, finishing speed, post-exercise recovery and reduced inflammation [37,50,53].

### 3.2. Details of Kefir Products Used in the Included Clinical Trials

Kefir is a fermented dairy product produced either with traditional kefir grains or defined commercial starter cultures. This definition is in line with international standards, including the Turkish Food Codex Fermented Dairy Products Directive (No: 2009/25) and the Codex Standard for Fermented Milks (CODEX STAN 243-2003) [67], which recognize kefir as a product fermented by lactic acid bacteria and yeasts derived from kefir grains [47,68]. Traditional grains contain a complex symbiotic community of bacteria and yeasts that produce a diverse range of bioactive metabolites and contribute to the characteristic flavor, texture, and potential health effects. Commercial starter cultures are designed to mimic these microbes for standardized, consistent production, but generally have lower microbial diversity [7,10,11,12]. This distinction is important when interpreting clinical trials, as differences in production can influence kefir’s functional properties. Table 3 summarizes the kefir products used in the 28 clinical trials included in this review, noting the production method for the kefir beverage (grains vs. starter cultures), microbial composition, and key characteristics. In some trials, information was not reported and is indicated as “details not reported.”

Table 3 presents the differences in kefir production that may have influenced study outcomes and functional properties. Across the 28 included clinical studies, kefir was produced using traditional grains in 8 studies [38,44,45,53,54,55,58,60] and commercial starter cultures in 12 studies [35,36,38,40,41,42,46,48,49,50,51,57]. Where Gupta et al. 2024 [38] reported the use of both kefir grains and starter culture, perhaps meaning an exopolysaccharide matrix with kefir microbiota. In 9 studies, the production method was not reported [37,39,43,47,52,56,59,61,62]. Most commercial kefirs provided 100–140 kcal per 237–250 mL serving, with larger servings or phase-specific formulations reaching 334–1000 kcal. Macronutrient content varied, with fat ranging from 2 to 7.3 g/250 mL, carbohydrates 4–15 g/250 mL, and protein 3–9 g/250 mL; milk-based controls generally had slightly higher protein. Calcium content was reported in a few studies, around 0.3 g/250 mL.

Lactic acid bacteria—including *Lactobacillus*, *Lacticaseibacillus*, *Lactococcus*, *Leuconostoc*, and *Streptococcus* genera—were consistently present across studies that reported microbial composition, while *Bifidobacteria* were reported in only three studies [35,40,41]. Yeast genera such as *Saccharomyces*, *Kluyveromyces*, *Kazachstania*, *Rhodosporidium*, and *Pichia* were reported in studies that provided a more comprehensive characterization of the microbial composition of kefir grains or starter cultures, rather than limiting analyses to lactobacilli [36,40,41,42,45,46,54,55,57,59,61]. Total microbial counts of kefir microbiotas mainly ranged between 10^8^ and 10^10^ cfu/mL. Several studies did not report microbial composition [37,38,39,44,49,50,51,52,56,58,60,62].

## 4. Discussion

In the review, 28 clinical trials were analyzed [35,36,37,38,39,40,41,42,43,44,45,46,47,48,49,50,51,52,53,54,55,56,57,58,59,60,61,62] that investigated the effects of kefir consumption on a broad range of health outcomes in humans. The studies included diverse populations, ranging from healthy individuals to patients with metabolic syndrome, non-alcoholic fatty liver disease (NAFLD), inflammatory bowel disease (IBD), osteoporosis, chronic functional constipation, and athletes, and focused on the influence of kefir on gastrointestinal health, metabolic, cardiovascular health, oral health outcome as well as lactose intolerance. Overall, kefir consumption was associated with various beneficial effects; however, the observed outcomes were dose-dependent, duration-dependent and the population profile was critical in determining the clinical efficacy.

### 4.1. Effects of Kefir Consumption on Gastrointestinal Health via Gut Microbiome Modulation

This section synthesizes findings from the clinical trials included in Table 2 and Table 3 of this review that specifically investigated the effects of kefir consumption on gastrointestinal health and gut microbiome modulation. Across these studies, kefir intake was associated with broadly consistent outcomes, including reductions in gastrointestinal symptoms [47,55,58], improved lactose tolerance [61], alleviation of constipation-related complaints [55] and modulation of the gut microbiota. Reported microbial changes included increased abundances of beneficial taxa such as *Lactobacillus*, *Bifidobacterium adolescentis*, *Akkermansia muciniphila*, and *Faecalibacterium prausnitzii*, which are commonly linked to anti-inflammatory effects and improved metabolic parameters [37,38,47]. The observed increases in taxa such as *Lactobacillus*, *Bifidobacterium*, *Akkermansia muciniphila*, and *Faecalibacterium prausnitzii* are important, as these microorganisms are well-known producers of short-chain fatty acids (SCFAs), particularly acetate and butyrate, which contribute to epithelial barrier integrity and immunomodulation. In the included clinical trials, improvements in gastrointestinal symptoms were also noted along with microbial shifts [37,47,55] and, in some studies, reductions in inflammatory markers such as CRP and TNF-α were observed [42,46]. Although SCFA concentrations were not measured in all clinical trials, the increase in SCFA-producing taxa and reduction in inflammatory parameters indicate that microbiota modulation may partly explain the beneficial metabolic and anti-inflammatory effects of kefir observed in these studies.

These findings are supported by previous reviews [10,70,71,72,73] which describe kefir as a complex fermented beverage containing a diverse consortium of lactic acid bacteria, acetic acid bacteria and yeasts, and a wide range of microbial metabolites and bioactive compounds, including exopolysaccharides, bioactive peptides, and organic acids; these collectively contribute to kefir’s capacity to modulate the intestinal microbiota, exert antimicrobial effects, enhance microbial diversity, and support the growth of beneficial taxa, thereby plausibly underlying the gastrointestinal benefits observed in the included clinical trials.

However, not all studies demonstrated consistent microbiota changes. For example, some trials reported no significant differences in overall microbial diversity, gut health or inflammatory markers following kefir consumption [38,59,60]. These findings suggest that the effects of kefir on gut microbiota may depend on baseline microbial composition, population characteristics, and intervention duration.

Despite these promising associations, it remains challenging to draw firm conclusions about kefir’s effects on a “healthy” human microbiome. The concept of a healthy microbiome is not universally defined, as individual gut microbiota compositions are highly personalized. However, certain core taxa are shared across humans [74,75,76]. Recently, the International Scientific Association for Probiotics and Prebiotics (ISAPP) published a new consensus document stating that gut health is defined as “a state of normal gastrointestinal function without active gastrointestinal disease and gut-related symptoms that affect quality of life” [77]. Another problem is high heterogeneity in study design, including kefir production, kefir composition and dosage, intervention duration, and participant characteristics, which further complicates interpretation. Although kefir consumption appears to support microbiota modulation and related metabolic and inflammatory outcomes in humans, further standardized, well-characterized clinical studies are required to clarify its precise effects and to improve frameworks for defining microbiome health [10,70].

Most studies assessing gut microbiota relied on 16S rRNA gene sequencing or culture-based approaches, as reflected by reporting of alpha-diversity indices such as Shannon and Chao1 [37,38,47]. Few studies incorporated functional analyses, and none of the reviewed trials applied comprehensive shotgun metagenomic or untargeted metabolomic approaches. This methodological variability may contribute to differences in reported findings and limits direct comparison of functional outcomes across studies.

Future research concerning kefir consumption, gastrointestinal health and intestinal microbiota modulation would benefit from the inclusion of shotgun metagenomic and metabolomic approaches [78,79], which could provide deeper insight into functional changes in the gut microbiome as well as the microbial metabolic activity, rather than relying solely on taxonomic shifts [80]. Such approaches would allow assessment of microbial pathways, metabolite production, and host–microbe interactions that may underlie the observed clinical effects of kefir. However, the application of these advanced techniques is currently limited by high costs, analytical complexity, and the need for specialized infrastructure [81,82], which may partly explain their limited use in clinical nutrition studies.

### 4.2. Effects of Kefir Consumption on Metabolic and Cardiovascular Health

Several RCTs included in this review [35,40,42,45,46,49,51,62] investigated the effects of kefir consumption on metabolic and cardiovascular health and found that kefir consumption is associated with beneficial effects on metabolic and cardiovascular health, particularly in individuals with overweight, metabolic syndrome, or non-alcoholic fatty liver disease (NAFLD). Kefir intake was linked to improvements in lipid profiles, reductions in blood pressure, and enhanced insulin sensitivity. Reported outcomes included decreases in LDL-cholesterol, triglycerides, homocysteine, fasting glucose, insulin, HOMA-IR, and inflammatory markers, alongside increases in HDL-cholesterol and apolipoprotein A1 (ApoA1). These improvements in lipid profile, insulin sensitivity, and inflammatory markers may be partially explained by kefir-induced alterations in the gut microbiota, including increased abundance of SCFA-producing taxa.

These findings are consistent with evidence from systematic reviews and meta-analyses, which report that kefir consumption can improve glycemic control, reduce insulin resistance, and lower inflammatory markers such as TNF-α and IL-6 across diverse adult populations [28,33,71]. These reviews also suggested that kefir is generally safe for consumption in healthy individuals and may confer cardiometabolic benefits; however, effects on lipid profiles, gut microbiota composition, and metabolic outcomes remain inconsistent across studies, highlighting the need for further well-designed human trials [7,32,70,83].

On the other hand, several studies reported no significant improvements in lipid profiles, glycemic parameters, or anthropometric outcomes compared with control groups [35,42,62]. These inconsistencies highlight the heterogeneity of study designs, kefir composition, and participant characteristics.

Previously published animal model studies have also demonstrated that traditional kefir has the potential to improve metabolic dysfunction associated with obesity. Traditional kefir was found to reduce body weight gain, plasma cholesterol levels, and hepatic triglycerides in mice. In contrast, commercial kefir showed no beneficial effect [84]. Additionally, kefir-derived exopolysaccharides have shown promising effects in experimental rats’ models of type 2 diabetes mellitus, suggesting potential nutraceutical applications [85].

Several other preclinical studies in animal models have shown beneficial effects of kefir consumption, showing reductions in oxidative stress, inflammation, and improvements in metabolic and cardiovascular parameters. In aged, stressed, hypertensive, NASH, and myocardial infarction animal models, kefir restored antioxidant enzyme activities, reduced lipid peroxidation, decreased pro-inflammatory cytokines (IL-6, TNF-α, TGF-β1), and limited fibrosis in liver, kidney, and heart tissues [86,87,88,89,90]. Systematic reviews of animal model studies also confirm that kefir consistently modulates immune and oxidative pathways, supporting its classification as a functional food with multi-system benefits for metabolic health promotion [73,91,92]. Although animal studies cannot be directly extrapolated to humans, their concordance with the clinical evidence reviewed here supports the conclusion that kefir consumption exerts beneficial effects on metabolic and cardiovascular health, while highlighting the need for further standardized human studies.

### 4.3. Other Effects of Kefir Consumption on Health

Among the RCTs included in this review, kefir consumption was associated with additional health benefits beyond gastrointestinal and cardiometabolic outcomes. One study [37] found that professional athletes consuming kefir showed an increase in *Akkermansia muciniphila* and *Faecalibacterium prausnitzii*, microbial taxa linked to improved energy metabolism and anti-inflammatory effects. Another study [53] found reduced inflammation after endurance training after kefir intake. Several studies [39,48,54,57] investigated oral health benefits and found reductions in *Streptococcus mutans* counts and improved periodontal indices. Additionally, one study [61] showed that kefir improved lactose digestion, likely mediated by β-galactosidase activity. These effects are likely driven by the live microbiota of kefir, particularly *Lactobacillus* spp. and yeasts, which can temporarily colonize the oral cavity, compete with pathogenic bacteria, and support lactose hydrolysis.

On the other hand, kefir did not demonstrate clear benefits in all clinical contexts. For example, some studies investigating chemotherapy-related outcomes or antibiotic-associated diarrhea reported no significant advantage compared with control interventions [58,59,60].

These findings demonstrate that the functional effects of kefir extend beyond gut microbiota modulation to include oral health, lactose digestion, and microbial contributions to systemic metabolic and anti-inflammatory outcomes. Consistent with these findings, other studies on fermented dairy products containing live beneficial microbes have also demonstrated reductions in oral pathogen load, improvements in dental health, and enhanced lactose tolerance in both adults and children [93,94,95,96].

### 4.4. Population-Specific Observations Due to Kefir Consumption

The studies included in this review cover a broad range of populations, which inevitably affects how their findings can be interpreted. Some trials were focused on individuals with metabolic conditions, such as non-alcoholic fatty liver disease, metabolic syndrome, elevated body mass index and dyslipidemia [35,40,42,44,45,46,49,51,52,62]. Other studies involved more clinically complex groups, for example, patients with Crohn’s disease or other inflammatory bowel diseases [47] and other various gastrointestinal diseases [55,56,58,59,61] or even critically ill patients [38,60]. At the same time, some interventions were tested on dental patients [39,48,57] and physically active or athletic cohorts [37,50,53].

These populations not only differ considerably in their metabolic status, inflammatory load, diet and physical activity, but also in gut microbiota composition [97,98,99]. It is therefore not surprising that the magnitude, and sometimes even the direction of the effects vary between studies. Rather than indicating inconsistency, this likely reflects population-specific mechanisms. The diversity of the included groups shows how widely kefir is being studied and highlights the need for caution when comparing results across such distinct settings [28,92]. Despite the heterogeneity of the study populations included in this review, meaningful improvements in metabolic, inflammatory, gastrointestinal, or microbiota-related outcomes were found following kefir consumption. This suggests that the potential benefits of kefir intake are not limited to a single clinical context.

### 4.5. Terminology and Mislabelling

A considerable number of authors have used terminology such as “probiotic kefir,” “probiotic microorganisms in kefir,” or “probiotic fermented milk” inappropriately [22,35,37,39,41,42,43,44,45,46,47,49,50,51,53,54,55,56,57,59,60,62,66,100,101]. According to the International Scientific Association for Probiotics and Prebiotics (ISAPP), kefir is classified as a fermented food and not as a probiotic product unless it contains well-defined, characterized strains at demonstrated health-benefit doses [15,16].

Although kefir consumption clearly leads to a broad range of clinically demonstrated health effects, researchers should avoid labeling kefir itself as “probiotic” or implying probiotic health claims without strain-specific evidence. Kefir grains contain a diverse and variable consortium of bacteria and yeasts, and the microbial composition differs substantially from standardized probiotic formulations. Therefore, the term “probiotic kefir” is scientifically inaccurate and potentially misleading. A similar discussion has been raised for other fermented foods, such as kimchi, where the question of whether it deserves probiotic status has been debated in the literature [102]. However, according to the International Scientific Association for Probiotics and Prebiotics (ISAPP), fermented foods should not be classified as probiotics unless they contain well-defined, strain-characterized microorganisms shown to confer a health benefit at an effective dose. In line with this position, kefir is more appropriately described as a fermented milk beverage that may exert probiotic-like effects rather than as a probiotic product.

On the other hand, several other studies have examined kefir fortified with additional probiotic strains, and such products may indeed confer health benefits; [22,36,66,101,103,104,105,106]; however, it remains unclear whether the observed effects are attributable to the added probiotic strains, the fermented kefir matrix itself, or a combination of both. Due to this, they were not included in the evidence synthesis, which focused on the health benefits of kefir consumption. For example, the study included in this review [36] investigated kefir and kefir with added probiotics, and the latter showed superior outcomes in depression and appetite regulation compared to kefir alone, suggesting potential synergistic effects. Studies that reported non-health-related outcomes (e.g., microbiota composition alone) [70] were also outside the predefined eligibility criteria.

### 4.6. Differences in Using Kefir Grains vs. Starter Cultures

The clinical studies included in this review included heterogeneous kefir interventions that differ substantially in daily dosage, duration of intake, and type of kefir preparation. While some kefir products were distributed directly by manufacturers, others were fermented under controlled laboratory or household conditions. Also, some kefir products were produced via fermentation using kefir grains [38,44,45,53,54,55,58,60] while others were made from starter cultures [35,36,38,40,41,42,46,48,49,50,51,57] and for some studies no data was given [37,39,43,47,52,56,59,61,62].

As highlighted by Vieira et al. [71], artisanal kefirs using traditional kefir grains and industrial kefirs produced from starter cultures differ in their bioactive compound profiles (exopolysaccharides, including kefiran, bioactive peptides, and organic acids, especially lactic acid), which can influence their functional potential [107,108]. This methodological diversity represents an important source of variability across trials and complicates the direct comparison of outcomes. Rather than indicating contradictory evidence, this heterogeneity is likely a consequence of the differences in kefir composition, fermentation conditions, and host-related factors [6,109,110]. Only one study included in this review [40] compared pitched kefir using traditional microorganisms and commercial kefir, finding differences in health benefits, where pitched kefir was more effective for cardiometabolic effects and commercial kefir was more effective in increasing TNF-alpha.

Although the studies included in this review that reported using kefir grains generally described a broader microbial community, several starter cultures also contained mixed bacterial and yeast consortia, similar to those in traditional kefir [10,109], the distinction in effectiveness was not necessarily obvious. Consequently, the available clinical evidence does not support firm conclusions regarding systematic differences in microbial composition or health effects between grain-based and starter-culture kefirs. Therefore, there is a need for standardized microbial characterization in future trials.

### 4.7. Dose-Dependent and Duration-Dependent Effects of Kefir Consumption

Findings from this review indicate that the beneficial effects of kefir consumption depended on both the daily volume of kefir consumed and the duration of intake. Short-term interventions (≤4 weeks) with moderate volumes (200–250 mL/day) were sufficient to improve gut microbiota diversity, stool frequency, and bowel satisfaction [37,38,55]. Whereas medium-term studies (6–12 weeks) with higher volumes (300–600 mL/day) demonstrated improvements in metabolic parameters, inflammatory markers, and appetite regulation [44,46,49,51]. These findings suggest that although gut-related effects may manifest relatively low volumes over short periods, systemic metabolic and inflammatory outcomes generally require higher volumes and longer durations of kefir intake.

Similarly, clinical studies using defined probiotic strains have found that short-term interventions (2 to 4 weeks) were sufficient to improve gastrointestinal symptoms, stool frequency, reflecting local gut microbiota modulation and dynamics [111,112]. In contrast, systemic metabolic, immunomodulatory or inflammation outcomes required longer intervention periods to achieve measurable changes in markers such as lipid profiles, inflammatory cytokines, or short-chain fatty acid production in human subjects [111,113,114,115]. Although it remains essential to distinguish kefir as a complex fermented food and probiotics as defined microbial interventions with strain-specific evidence, the parallels in dosage and duration effects between kefir and probiotics emphasize the general principles of microbial interventions.

### 4.8. Practical Implications of Kefir Consumption

Kefir is also increasingly becoming recognized as a functional fermented milk beverage that can support human health on multiple levels [23,31], and based on the evidence from this review, the potential benefits include gastrointestinal health, metabolic regulation, and modulation of inflammatory processes. While general recommendations can be given for daily consumption (200–600 mL/day depending on duration and targeted outcome), more research is needed to determine the optimal dose and composition. However, further research is required to define optimal doses and microbial compositions for specific clinical applications.

From a clinical nutrition perspective, the current evidence suggests that milk-based kefir may be incorporated as part of a balanced diet, particularly in individuals with mild metabolic disturbances, gastrointestinal complaints, or lactose intolerance. However, due to heterogeneity in kefir composition, dosage, and study populations, kefir should not be considered a therapeutic substitute for established medical treatments. Healthcare professionals should interpret the available evidence cautiously and consider individual patient characteristics, including baseline metabolic status, dietary habits, and tolerance to fermented dairy products.

These findings also have implications for clinical nutrition practice, dietary guidelines, and the development of functional fermented beverages. Currently, the lack of standardized microbial enumeration and detailed reporting of nutritional composition limits the comparability of products and hinders the establishment of dose–response relationships [6]. Routine measurement and declaration of microbial counts and composition by manufacturers would enable more systematic clinical research, allowing meaningful comparisons across studies [110,116]. Such standardization is essential for high-quality systematic reviews and meta-analyses, which in turn could help identify effective doses and formulations for specific health outcomes [116]. Furthermore, consistent with ISAPP recommendations [15,16], it is important to clearly distinguish fermented foods from probiotic products unless strain-specific microorganisms are defined and shown to confer a health benefit at an effective dose.

Although kefir shows promising health benefits, it does not meet the formal definition of a probiotic. Greater transparency in labeling, including reporting microbial composition and viable counts where feasible, would support both clinical research and informed consumer choice. Overall, the available clinical evidence supports the inclusion of milk-based kefir as a functional fermented food within a diverse dietary pattern. However, further well-designed and adequately powered clinical trials using standardized kefir preparations are required before specific health claims or formal dietary recommendations can be established.

### 4.9. Limitations and Recommendations

This review has several limitations, mainly connected to the properties of kefir. Blinding is particularly challenging in kefir research due to its characteristic taste, aroma, texture, and effervescence. Most trials were open-label or single-blind, which may introduce expectation bias. Only three studies included in this review reported double-blind designs: da Silva Ghizi et al. [44] reported identical packaging without clear sensory matching, while Bekar et al. [56] did not specify methods. The most rigorously described approach was Merenstein et al. [59], which used heat-inactivated, matched flavoring, appearance, and packaging to achieve perceptual equivalence. These organoleptic constraints should be considered when interpreting clinical evidence. Future trials should aim to improve blinding or use carefully matched sensory controls to minimize expectation bias and strengthen methodological rigor.

A major methodological limitation is that the included studies also differed in kefir production methods, microbial composition, daily dose, and duration, which complicates direct comparisons and interpretation of findings. Standardized reporting of microbial content, daily intake, intervention duration and differentiation between grain-fermented and starter-culture kefir is recommended. Future studies should investigate longer intervention periods, larger sample sizes, and more homogeneous populations to enhance comparability and strengthen conclusions.

Besides these two major limitations, sample sizes of clinical trials were often small, and populations were heterogeneous, ranging from healthy individuals—such as athletes—to patients with inflammatory bowel disease, metabolic syndrome, or dental conditions, limiting generalizability. Assessed outcomes were also heterogeneous, including metabolic and inflammatory parameters, gut microbiota modulation, cognitive and oral health, as well as exercise-related parameters. Several studies were short-term, preventing the evaluation of long-term effects and trials not published in English were not included [117,118].

A further limitation is that microbiota analyses were largely limited to compositional profiling, with little assessment of microbial function. This restricts the interpretation of how kefir may influence metabolic or inflammatory pathways.

## 5. Conclusions

Kefir consumption appears to offer broad health benefits, including support for the gut microbiota, metabolic and immune function, and positive effects on oral, cognitive, and exercise-related outcomes. Evidence from 28 clinical trials, including recent randomized studies, shows these benefits across diverse populations, although their magnitude can vary depending on microbial composition, daily dose, and duration of kefir consumption. Traditional grain-based kefirs, with higher microbial diversity, may produce stronger effects than starter culture-based versions, though both forms were found to be beneficial.

The heterogeneity across the studies points to the need for more standardized research, including detailed reporting of microbial composition and viable counts, consistent dosing, longer interventions, and, where feasible, double-blind designs with matched sensory controls. While this review may not have included every published study on kefir consumption, the overall pattern of findings supports the conclusion that kefir is a safe, functional fermented milk beverage with important health potential. Continued high-quality clinical studies are needed to clarify optimal consumption patterns and the mechanisms behind kefir’s health-promoting effects.

## Figures and Tables

**Table 1:** Research strategy containing inclusion criteria and search limits.

Databases	PubMed, Web of Science, Scopus and Manual Search
Search strategy:	(“kefir” OR “milk kefir” OR “fermented milk”) AND (“Clinical Trial” OR “Randomized Controlled Trial” OR “RCT” OR “clinical study”) AND (“health benefits” OR “health effects”)
Types of research:	Human clinical trials investigating kefir consumption.
Language:	Publications in English.
Exclusion criteria:	Studies that utilized kefir with added probiotics, synbiotics or prebiotics were excluded to isolate only the influence of the kefir milk beverage. Studies that utilized water kefir. Publications in languages other than English.
Timeframe:	Up to 30 August 2025.

**Table 2:** Characteristics and main outcomes of 28 human clinical trials investigating kefir as a fermented milk product (in descending chronological and alphabetical order).

Reference	Study Design	Aim	Participants (Completed)	Intervention Protocol	Main Findings After Kefir Consumption
Mohammadi et al., 2025 [35]. Iran.	Two-arm parallel-group RCT	To evaluate the effects of kefir drink on liver aminotransferases, high-sensitivity CRP and metabolic parameters in patients with NAFLD	72 patients with NAFLD, aged 42.87 ± 10.67 years. n = 36 kefir group, n = 36 control group.	Kefir intervention: 2 × 250 mL kefir/day + dietary plan. Control: dietary plan. Duration: 8 weeks.	No significant effect on liver aminotransferases and metabolic indicators in patients with NAFLD. Significant effect on HDL-C, FBS and fat-free mass in patients with NAFLD. Both groups exhibited significant differences in ALT, AST.
Noori et al., 2025 [36] Iran.	Two-arm parallel-group RCT	To evaluate the effects of kefir drink with or without added probiotics on depression, appetite, oxidative stress, and inflammatory parameters in overweight and obese elderly individuals.	67 overweight males, aged over 65 years. n = 32 kefir group, n = 36 kefir with probiotics group.	Kefir interventions: A: 240 mL kefir/day. B: 240 mL kefir with added probiotics/day. Duration: 8 weeks.	No significant differences in oxidative stress or inflammatory parameters between groups. Significant differences between groups in favor of kefir with added probiotics in depression and appetite scores.
Öneş et al. 2025 [37]. Turkey.	Two-arm parallel-group RCT	To assess gut microbiome, body composition, and performance after kefir consumption of professional female soccer players.	21 professional female soccer players, aged 18–29 years. n = 12 kefir group, n = 9 control group.	Kefir intervention: 200 mL kefir/day. Control: no treatment. Duration: 4 weeks (28 days).	Increased gut microbial diversity in kefir group (Shannon, Chao1 indexes) in favor of *Akkermansia muciniphila* and *Faecalibacterium prausnitzii* (regulators of energy metabolism with anti-inflammatory effects. Higher athletic performance variables (VO_2_ max and finishing speed due to higher abundance of SCFA-producing bacteria in kefir group.
Gupta et al. 2024 [38]. United States of America.	Open-label, single-arm phase I	To assess the safety and tolerance of kefir in critically ill patients.	54 critically ill adults.	Kefir intervention: 60 mL kefir/day, then 120 mL/day after 12 h, then 240 mL/day). Control: no treatment. Duration: between 3 days and 4 weeks (until ICU discharge).	Kefir is well-tolerated, no cases of bacteriemia. No significant increase in gut microbial alpha diversity (Shannon index) in either group. Significantly improved GMWI in kefir group.
Şahin et al. 2024 [39]. Turkey.	Three-arm parallel-group RCT	To assess the effect of kefir and probiotic tablets combined with IPT on oral microbiota composition and gingival health in participants with periodontitis.	36 participants with stage 1 and 2 periodontitis, aged 18–70 years. n = 12 kefir group, n = 12 probiotic group, n = 12 control group.	Intervention: 250 mL kefir/day + IPT. Comparator: Probiotic tablets + IPT. Control: IPT alone. Duration: 14 days.	Improved PI, GingI, BOP, PPD, CAL after 3 months in kefir group (comparable to probiotic group and control). Decreased fecal *Tannerella forsythia* in kefir group (comparable to the probiotic group and control group). Nonsignificant reduction in fecal *Porphyromonas gingivalis* and *Treponema denticola* in kefir group.
Bourrie et al. 2023 [40]. Ireland.	Crossover pilot RCT	To compare pitched kefir (with traditional microorganisms) and commercial kefir (without them) regarding LDL cholesterol, inflammatory markers, and endothelial function in adult males with slightly elevated LDL-C.	21 adult males with slightly elevated LDL-C (3.2–4.9 mmol/L), aged 18–65 years. n = 21–crossover.	Intervention: A: 2 × 350 g commercial kefir/day. B: 2 × 350 g pitched kefir/day. Duration: intervention: 2 × 4 weeks; washout: 4 weeks (12 weeks total).	Increase in TNF-α with commercial kefir. Greater reduction in LDL-C, sICAM-1, sVCAM-1, CRP, IL-8 and TNF-α with pitched kefir. No effects on total cholesterol, HDL-C, triglycerides, glucose, or insulin in either groups. Microbial composition identified as a key factor for kefir’s cardiometabolic benefits.
Cannavale et al. 2023 [41]. United States of America.	Single-blinded, crossover RCT	To evaluate the influence of kefir vs. control on memory, gut microbiota, mood, and stress in healthy adults.	26 healthy adults free of gastrointestinal and mental illness, aged 25–45 years. n = 26–crossover.	Intervention: 237 mL kefir/day. Control: Lactose-free 1% low-fat milk. Duration: intervention: 2 × 4 weeks; washout: 2–4 weeks (10–12 weeks total).	Statistically significant memory improvements in kefir group. No significant changes in UFC, depression, anxiety in kefir group. Lower stress scores. Increased fecal lactobacilli and bifidobacteria and decreased *Phascolarctobacterium* in kefir group. No correlation between microbiota changes and memory improvements.
Bellikci-Koyu et al. ** 2022 [42]. Turkey.	Two-arm parallel-group RCT	To evaluate the effects of kefir vs. control on metabolic syndrome components, biochemical markers, inflammation, anthropometrics, and blood pressure in patients with metabolic syndrome.	62 adults (18–65 years) with MS. n = 31 kefir group, n = 31 control group.	Intervention: 180 mL kefir/day. Control: 180 mL unfermented milk/day. Duration: 12 weeks.	Significant increase in ApoA1 in kefir group. Significant reduction in homocysteine and cytokines (TNF-α, IL-6, IL-10, IFN-γ) vs. baseline within kefir group. No significant differences in glucose, insulin, HbA1c, HOMA-IR, lipids, cytokines, or blood pressure.
Caferoglu et al., 2021 [43]. Turkey.	Crossover RCT	To determine whether kefir, consumed with low- or high-glycemic index meals, affects appetite and subsequent food intake in healthy females.	24 healthy females, aged 21–24 years.	Intervention: 200 mL kefir + low-GI-meal or high-GI-meal. Control: 200 mL milk + low-GI-meal. Duration: intervention: 3 × 1 day; washout: 7 days (25 days total).	No differences in appetite scores and voluntary energy intake between test meals. Comparable palatability ratings between test meals, with higher overall palatability for high-GI kefir. Appetite-regulating effect of kefir added to a high-GI meal, producing postprandial responses like a low-GI meal.
Da Silva Ghizi et al., 2021 [44]. Brazil.	Double blind two-arm parallel-group RCT	To examine the effect of kefir on anthropometric and physiological parameters in patients with metabolic syndrome (MS).	48 patients with MS. n = 24 kefir group (42 ± 14 years), n = 24 control group (44 ± 10 years).	Intervention: kefir. Control: curd (mentioned below). Men: 1.6 mL/kg body weight/day. Women: 1.9 mL/kg body weight/day. Duration: 12 weeks.	Improved blood pressure, fasting glycemia, lipid profile in women. Reduced predicted ten-year cardiovascular risk. Unchanged anthropometric parameters despite kefir intake. Potential beneficial effects of regular kefir intake on metabolic syndrome management.
Jenko Pražnikar et al., 2020 [45]. Slovenia.	Open-label, crossover RCT	To investigate effects of kefir vs. control on serum zonulin, inflammatory markers, lipid profile, glucose, anthropometry, and mood in overweight adults.	28 overweight asymptomatic adults, aged 30 and 60 years. n = 28–crossover.	Intervention: 300 mL kefir/day. Control: 300 mL milk/day. Duration: intervention: 2 × 21 days; washout: 7 days (8 weeks total).	Significant reduction in serum zonulin and appetite perceptions in kefir group. No significant effects on CRP, adiponectin, lipid profile, glucose, or anthropometry. Positive effect on mood and slightly reduced negative mood in kefir group.
Bellikci-Koyu et al. ** 2019 [46]. Turkey.	Parallel-group, two-arm RCT	To assess the effects of probiotic kefir vs. control on anthropometric measurements, glycemic control, lipid profile, blood pressure, and inflammatory markers in adults with metabolic syndrome (MS).	22 adults with MS, aged 18–65 years. n = 12 kefir group, n = 10 control group.	Intervention: 180 mL kefir/day. Control: 180 mL unfermented milk/day. Duration: 12 weeks.	Significant decrease in fasting insulin, HOMA-IR, TNF-α, IFN-γ, SBP and DBP in kefir group. No significant differences in anthropometry. Slight reduction in body weight and fat mass. Significant increase in the phyla fecal Actinomycetota *; other phyla change insignificant. No changes in alpha or beta diversity and no adverse effects reported.
Yılmaz et al., 2019 [47]. Turkey.	Two-arm parallel-group, open-label RCT	To investigate the effects of kefir vs. control on fecal microbiota and symptoms of patients with IBD.	45 adults with IBD (Crohn’s disease and ulcerative colitis). n = 25 kefir group, n = 20 control group.	Intervention: 2 × 200 mL kefir/day. Control: no treatment. Duration: 4 weeks.	Significant improvement of abdominal pain, bloating, and QOL in kefir group. Increased fecal *Lactobacillus* spp. and *Lentilactobacillus kefiri* in kefir group. Improved Hgb and decreased ESR and CRP in participants with Crohn’s disease in kefir group.
Alp et al., 2018 [48]. Turkey.	Parallel group, three-arm RCT	To examine inhibitory effects of kefir and probiotic toothpaste in orthodontic patients.	45 orthodontic patients, aged 12–17 years. n = 15 kefir group, n = 15 toothpaste group, n = 15 control group.	Intervention: 2 × 100 mL kefir/day, Comparator: 2 × brushing with probiotic toothpaste/day. Control: regular tooth care. Duration: 6 weeks.	Decreased salivary *Streptococcus mutans* and *Lactobacillus* levels in the kefir and toothpaste groups vs. control. Increased salivary buffer capacity in the toothpaste group vs. kefir and control. Unchanged salivary flow rate across groups.
Fathi et al., *** 2017 [49]. Iran.	Three-arm, parallel group RCT	To determine the lipid-lowering effects of kefir in overweight or obese premenopausal women.	75 overweight or obese premenopausal women, aged 25–45 years. n = 25 kefir group, n = 25 milk group, n = 25 control group.	Intervention: 2 × 300 mL kefir/day + WMD, Comparator: 2 × 300 mL low fat milk/day + WMD, Control: WMD. Duration: 8 weeks.	Significant decrease in lipid parameters total cholesterol, LDLC, Non-HDLC, total cholesterol/HDLC, and LDLC/HDLC in kefir group. No significant changes in anthropometrics or dietary intake.
Gölünük et al., 2017 [50]. Turkey.	Three-arm, parallel group RCT	To determine the effects of kefir and boza beverages on blood values of aerobic exercisers (AE).	36 males, aged 18–25 years. n = 12 kefir group, n = 12 boza group, n = 12 control group.	Intervention: 300 mL kefir/day + 1 h AE. Comparator: 300 mL boza/day + 1 h AE Control: 1 h AE. Duration: 15 days.	Significant reductions in ALT and TOS in the kefir group. Significant reductions in triglycerides and VLDL and changes in chloride and creatine levels in boza group. No significant changes in blood parameters in the control group.
Fathi et al., *** 2016 [51]. Iran.	Three-arm, parallel group RCT	To determine the weight-reducing effects of kefir vs. control of overweight or obese premenopausal women.	75 overweight premenopausal women, aged 25–45 years. n = 25 kefir group, n = 25 milk group, n = 25 control group	Intervention: 2 × 300 mL kefir/day + WMD, Comparator: 2 × 300 mL low fat milk/day + WMD, Control: WMD. Duration: 8 weeks.	Greater reduction in weight, BMI, and waist circumference for both treatment groups. Higher dietary calcium intake in both treatment groups.
Kong et al., 2015 [52]. United States of America.	Crossover RCT with 3-phase design	To determine GI, II, and SI indices of three kefir types compared with glucose or white bread in healthy adults with elevated BMI.	10 healthy adults with BMI 22–24 kg/m^2^ aged 22–27 years. n = 10–crossover.	Intervention: Phase 1: 546 mL Strawberry & 548 mL orange kefir, phase 2: 667 mL plain—and phase 3: Three kefirs (331–479 mL). Controls: Phases 1–2: 50 g or 25 g dextrose solutions; phase 3 white bread. Duration: at least 4 days washout.	Significantly lower GI of all kefirs compared with glucose. Il values of kefirs comparable to white bread. SI values not exceeding those of white bread. Low-to-moderate GI classification of kefir with high II but no superior satiety effect relative to bread
O’Brien et al., 2015 [53]. United States of America.	Four-arm parallel group clinical trial	To determine whether kefir vs. control enhances the positive effects of endurance training (ET) on fitness, body composition, and CRP in healthy adults.	67 healthy adults, aged 18–24 years. n = 13 kefir group 1, n = 21 kefir group 2, n = 10 control group 1, n = 21 control group 2.	Intervention: Group 1: 2 × 454 g kefir/week + ET, Group 2: 2 × 454 g kefir/week. Controls: Group 1: 2 × 454 g lactaid milk/week + ET, Group 2: 2 × 454 g lactaid milk/week, Duration: 15 weeks.	Improved recovery and reduced inflammation by attenuating the increase in CRP in kefir group. No observed body composition differences in any groups. Kefir well accepted; suitable for lactose-intolerant athletes.
Ghasempour et al., 2014. [54] Iran.	Crossover RCT	To compare the inhibitory effects of kefir drink and NaF rinse on *Streptococcus mutans* counts in saliva of healthy adults.	22 healthy adults, aged 22–32 years. n = 11 kefir group, n = 11 control group.	Intervention: 100 mL kefir/day. Control: NaF rinse (0.05%). Duration: intervention: 2 × 2 weeks; washout: 4 weeks (8 weeks total).	Significant reductions in salivary *Streptococcus mutans* counts with both kefir and NaF. Comparable salivary reductions between kefir and NaF. Unchanged salivary pH in kefir group. Potential use of kefir as an alternative to NaF rinse for caries prevention.
Turan et al., 2014 [55]. Turkey.	Single-arm pilot trial	To evaluate the effects of kefir consumption on stool frequency, stool consistency, straining, laxative use, and bowel satisfaction in patients with chronic functional constipation.	20 adults with functional constipation, aged 27–78 years, with functional constipation. n = 10 slow transit group, n = 10 normal transit group.	Intervention: 2 × 250 mL kefir/day. Control: none. Duration: 4 weeks.	Significant increase in stool frequency, stool consistency and bowel satisfaction. Significant decrease in laxative use. Significantly shortened colonic transit time (slow-transit group). No serious adverse events.
Bekar et al., 2011 [56]. Turkey.	Double blind two-arm, parallel group RCT	To examine the effect of adding kefir to standard triple therapy on *Helicobacter pylori* eradication and treatment tolerance.	82 adults’ patients with dyspeptic symptoms and *H. pylori* infection. n = 46 kefir group, n = 36 control group.	Intervention: 2 × 250 mL kefir/day + triple therapy. Control: 2 × 250 mL milk containing placebo + triple therapy. Duration: 14 days.	Higher *Helicobacter pylori* eradication rate in kefir group. Lower and milder adverse effects (diarrhea, headache, nausea, abdominal pain) in kefir group. Improved *Helicobacter pylori* eradication and reduced side effects in kefir group alongside triple therapy.
Cogulu et al., 2010 [57]. Turkey.	Three-arm, parallel group RCT	To determine the effects of kefir on salivary counts of *Streptococcus mutans* and *Lactobacillus* spp. in young adults.	104, aged 20–27 years. n = 35 kefir group, n = 35 milk group, n = 34 control group.	Intervention: A: 100 mL kefir/day, B: 200 mL kefir/day, Control: 100 mL milk/day. Duration: 3 weeks.	Statistically significant reductions in both salivary mutans streptococci and lactobacilli in group with higher kefir intake compared to baseline.
Can et al., 2009 [58]. Turkey.	Two-arm, parallel group RCT	To determine the effect of kefir on the prevention of gastrointestinal complaints and on QOL of patients with colorectal cancer.	37 colorectal cancer patients, mean age 54.3 ± 12.8 year. n = 17 kefir group, n = 20 control group.	Intervention: 2 × 250 mL kefir/day. Control: no treatment. Duration: one week during each CT cycle (six cycles total).	Reduced sleep disturbances in kefir group. More treatment-related gastrointestinal complaints in kefir group following chemotherapy. No difference in QOL and prevention of gastrointestinal complaints.
Merenstein et al., 2009 [59]. United States of America.	Double-blinded, two-arm, parallel group placebo-controlled RCT	To examine the role of commercially available kefir in preventing AAD in children undergoing antibiotic treatment.	125 children aged 1–5 years presenting to primary care physicians. n = 61 kefir group, n = 64 control group.	Intervention: 75–150 mL lime-flavored kefir/day. Control: 75–150 mL heat-inactivated lime-flavored kefir/day. Duration: 10 days.	No significant differences in diarrhea, vomiting, abdominal pain, constipation, or other symptoms (e.g., runny nose, cough, fever, irritability). Reduction in AAD-related symptoms and improved overall health ratings were reported in both groups.
Topuz et al., 2009 [60]. Turkey.	Two-arm, parallel group RCT	To investigate the effect of kefir on chemotherapy-induced mucositis and systemic immune response (IL-1β, IL-6, TNF-α) of colorectal cancer patients.	37 colorectal cancer patients (Stage II–IV). n = 17 kefir group, n = 20 control group.	Intervention: 2 × 250 mL kefir (as oral lavage then swallowed)/day. Control: 2 × oral lavage with saline solution/day. Duration: 5 days per CT-cycle.	No kefir-related advantage in mucositis incidence or severity and limited antimicrobial activity of kefir. No significant change in mucositis progression with kefir. No effect on IL-1β, IL-6, or TNF-α levels.
Hertzler et al., 2003 [61]. United States of America.	Crossover RCT	To determine whether kefir improves lactose digestion and tolerance of healthy adults with confirmed lactose maldigestion.	15 healthy adults with confirmed lactose maldigestion, aged 20–34 years.	Intervention: (providing 20 g lactose): 508 g flavored kefir, 378 g plain kefir, 519 g flavored yogurt, 428 g plain yogurt. Control: 407 g milk, 2% fat. Duration: 1 day each.	Reduced breath hydrogen and peak hydrogen after kefir and yogurt. Decreased flatulence with all kefir and yogurt (less with flavored kefir). Minimal, comparable abdominal pain and diarrhea across treatments. Presence of β-galactosidase activity in kefir and yogurt. Improved lactose digestion and tolerance with kefir (plain > flavored).
St-Onge et al., 2002 [62]. Canada.	Crossover RCT	To determine whether kefir supplementation alters plasma total cholesterol, HDL-C, LDL-C, triglycerides, and fatty acid levels in mildly hypercholesterolemic men.	13 non-smoking mildly hypercholesterolemic men with BMI 26–38 kg/m^2^, aged 27–61 years.	Intervention: 500 mL kefir/day. Control: 500 mL milk/day. Duration: intervention: 2 × 4 weeks; washout: 4 weeks (12 weeks total).	Comparable plasma lipid profiles after kefir and milk. Similar cholesterol synthesis rates with kefir and milk. Increased fecal SCFAs with both treatments, with possible modulation of gut microbiota and SCFA production in kefir group. Higher fecal bacterial content with kefir.

* Actinomycetota (previously Actinobacteria) [65]; **, *** Results from the same clinical trial were reported in two separate publications: ** [42,46] *** [49,51]. Abbreviations: AAD: Antibiotic-associated diarrhea; AE: aerobic exercise; ALT: Alanine transaminase; ApoA1: Apolipoprotein A1; AST: Aspartate transaminase; BMI: Body mass index; BOP: Bleeding on probing; CAL: Clinical attachment level; Chao1: Estimator of species richness in microbiome samples; CRP: C-reactive protein; DBP: Diastolic blood pressure; ESR: Erythrocyte Sedimentation Rate; ET: Endurance Training; FBS: Fasting blood sugar; GingI: Gingival index; GI: Glycemic index; HbA1c: Glycated hemoglobin; GMWI: Gut Microbiome Wellness Index; Hgb: Hemoglobin; HDL-C: High-density lipoprotein cholesterol; HOMA-IR: Homeostasis Model of Assessment Insulin Resistance; IBD: Inflammatory Bowel Disease; ICU: Intensive care unit; IFN-γ: Interferon-gamma; IL-1β: Interleukin 1 beta; IL-6: Interleukin 6; IL-8: Interleukin 8; IL-10: Interleukin 10; II: Insulinemic indices; IPT: Initial periodontal treatment; LDL: Low-density lipoprotein; LDL-C: Low-density lipoprotein cholesterol; Low-GI-meal: breakfast with low glycemic index; High-GI: breakfast with high glycemic index; MS: Metabolic syndrome; NaF: Sodium fluoride; NAFLD: Non-alcoholic fatty liver disease; PI: Plaque index; PPD: Probing pocket depth; QOL: Quality of Life; RCT: Randomized controlled trial; SBP: Systolic blood pressure; SCFA: Short chain fatty acid; sICAM-1: Secretory Intercellular Adhesion Molecule 1; SI: Satiety indices; sVCAM-1: Secretory Vascular Cell Adhesion Molecule 1; TOS: Total Oxidant Status; Triple therapy: lansoprazole 30 mg, amoxicillin 1000 mg, clarithromycin 500 mg; VO_2_: Volume of oxygen; VLDL: Very low-density lipoprotein; vs.: versus.

**Table 3:** Characteristics of kefir interventions used in the 28 included clinical studies.

Reference	Dose; duration; Control *	Kefir and Comparator Production	Nutritional Composition	Microbial Composition **
Mohammadi et al. 2025 [35].	Kefir 500 mL/day; 8 weeks; dietary plan.	Fars Pegah Co. (Fars, Iran): commercial kefir: starter cultures.	250 mL kefir: 118 kcal, CHO 10 g, fat 5 g, protein 8 g, Ca 0.3 g.	2 × 10^9^ cfu bacteria (including lactobacilli and bifidobacteria)/250 mL kefir.
Noori et al. 2025 [36].	Kefir 240 mL/day; 8 weeks; kefir with probiotics.	Pegah Company (Fars, Iran): commercial kefir: starter cultures ± added probiotics.	Details NR.	Kefir starter: LAF4 and *Kl. marxianus*. Kefir with added probiotics: 3 × 10^9^ cfu *L. helveticus* R0052 and *B. longum* R0175 each/250 mL kefir.
Öneş et al. 2025 [37].	Kefir 200 mL/day; 4 weeks; no treatment.	Commercial kefir (Details NR).	250 mL kefir: 140 kcal, fat 7.3 g, CHO 4.2 g, protein 9.2 g.	Details NR.
Gupta et al. 2024 [38].	Kefir 60–240 mL/day; ≤4 weeks; no treatment.	Lifeway Foods (Morton Grove, IL, USA): commercial kefir: kefir grains and starter cultures	240 mL kefir: 140 kcal, fat 7.3 g, CHO 4.2 g, protein 9.2 g.	2.5–3.0 × 10^10^ cfu of active cultures. Details NR.
Şahin et al. 2024 [39].	Kefir 250 mL/day; 14 days; active control.	Atatürk Orman Çiftliği (Ankara, Turkey): commercial kefir. Probest Defense Abdi İbrahim (Istanbul, Turkey): probiotics.	Details NR.	Details NR.
Bourrie et al. 2023 [40].	Kefir (commercial) 700 g/day; 12 weeks (crossover); pitched kefir.	Liberte (Quebec, OC, Canada): commercial kefir: starter cultures.	700 mL kefir: 200 kcal. Other details NR.	Kefir: *L. lactis*, *L. rhamnosus*, *St. diacetylactis*, *L. plantarum*, *L. casei*, *Sacch. florentinus*, *Lu. cremoris*, *B. longum*, *B. breve*, *L. acidophilus*, *B. lactis*, *L. reuteri* (8.0 × 10^6^ cfu/mL). Pitched kefir: *A. pasteurianus*, *Lc. lactis*, *Lu. mesenteroides*, *L. kefiranofaciens*, *L. kefiri*, *P. fermentans*, *Sacch. cerevisiae*, *Ka. unispora*, *Kl. marxianus*.
Cannavale et al. 2023 [41].	Kefir 237 mL/day; 10–12 weeks (crossover); milk.	Commercial kefir: starter culture. Details NR.	237 mL kefir/milk: 100 kcal, fat 2.5 g/2 g, CHO 11 g/12 g, protein 8 g/11 g.	*L. lactis*, *L. rhamnosus*, *St. aiacetylactis*, *L. plantarum*, *L. casei*, *Sacch. florentinus*, *Lu. cremoris*, *B. longum*, *B. breve*, *L. acidophilus*, *B. lactis*, *L. reuteri.*
Bellikci-Koyu et al. 2022 [42].	Kefir 180 mL/day; 12 weeks; unfermented milk.	Prepared by authors: starter culture from Danisco (Olsztyn, Poland).	Details NR.	*Lc. lactis*, *Lc. cremoris*, *Lc. diacetylactis*, *Lu. mesenteroides*, *L. kefyr*, *Sacch. unisporus*, *Kl. marxianus.*
Caferoglu et al. 2021 [43].	Kefir 200 mL/day; 25 days (crossover); milk.	Details NR.	200 mL: kefir/milk—kcal 524/526; fat 6.8/6.3 g; CHO 9.9/10 g; protein 5.8 g. Ca 0.24 g.	10^7^ cfu bacteria (including lactobacilli and streptococci).
Da Silva Ghizi et al. 2021 [44].	Kefir 1.6–1.9 mL/kg/day; 12 weeks; curd.	Prepared by authors: kefir grains from Federal University of Viçosa, Brazil.	Details NR.	Details NR.
Jenko Pražnikar et al. 2020 [45].	Kefir 300 mL/day; 8 weeks (crossover); milk.	Kele & Kele (Logatec, Slovenia): commercial kefir: kefir grains.	100 g kefir/milk: 57/64 kcal, fat 3.2 g/3.5 g, CHO 3.9 g/4.7 g, protein 3.2 g/3.3 g, Ca: 0.12 g.	*L. parakefiri*, *L. kefiri*, *L. kefiranofaciens*, cocci. *Kl. marxianus*, *Ka. exigua*, *Rghodo. kratochvilovae.*
Bellikci-Koyu et al. 2019 [46].	Kefir 180 mL/day; 12 weeks; unfermented milk.	Prepared by authors: starter culture from Danisco (Olsztyn, Poland).	Details NR.	*Lc. lactis*, *Lc. cremoris*, *Lc. diacetylactis*, *Lu. mesenteroides*, *L. kefyr*, *Kl. marxianus*, *Sacch. unisporus*.
Yılmaz et al. 2019 [47].	Kefir 400 mL/day; 4 weeks; no treatment.	Details NR.	Details NR.	2.0 × 10^10^ cfu/mL viable *Lactobacillus* bacteria.
Alp et al. 2018 [48].	Kefir 200 mL/day; 6 weeks; active control	Atat€urk Orman Ciftligi (Ankara, Turkey): commercial kefir: starter culture.	Details NR.	*Lc. lactis*, *Leuconostoc* spp., *Lactobacillus* spp., *St. thermophilus*, yeasts.
Fathi et al. 2017 [49].	Kefir 600 mL/day; 8 weeks; milk/diet.	Fars Pegah Co. (Fars, Iran): commercial kefir: starter cultures.	250 mL kefir/milk: 118/116 kcal, CHO 10/12 g, fat 5/4 g, protein 8 g, Ca 0.3 g.	Details NR.
Gölünük et al. 2017 [50].	Kefir 300 mL/day; 15 days; exercise.	Atatürk Orman Çiftliği (Ankara, Turkey): commercial kefir: starter culture.	Details NR.	Details NR.
Fathi et al. 2016 [51].	Kefir 600 mL/day; 8 weeks; milk/diet.	Fars Pegah Co. (Fars, Iran): commercial kefir: starter cultures.	250 mL kefir/milk: 118/116 kcal, CHO 10/12 g, fat 5/4 g, protein 8 g, Ca 0.3 g.	Details NR.
Kong et al. 2015 [52].	Kefir phase-specific (546 mL, 667 mL, <479 mL); washout 4 days (crossover); energy-matched.	Fisherbrand Sun-Dex and Fisher Health Care, (Houston, TX, USA): commercial kefir: detail NR.	Phase I kefirs (546 mL): 396–481 kcal, CHO 50 g, fat 5–19 g, protein 32–33 g. Phase II kefir (667 mL): 334 kcal, CHO 25 g, fat 6 g, protein 39 g. Phase III kefirs (273–479 mL): 1000 kcal, CHO 18–30 g, fat 3–9 g, protein 16–28 g.	Details NR.
O’Brien et al. 2015 [53].	Kefir 908 g/week; 15 weeks; milk.	Prepared by authors: kefir grains from Cultures for Health, Sioux Falls, SD, USA).	908 g kefir/control: 220.16 kcal, CHO 66.56 g, fat 5.4 g, protein 10.24 g, Na 71.68 mg.	10^9^–10^10^ cfu lactic acid bacteria/454 g, 10^7^–10^8^ cfu yeasts/454 g.
Ghasempour et al. 2014. [54].	Kefir 100 mL/day; 8 weeks (crossover); active control.	Homemade kefir: Iranian kefir grains.	Details NR.	*L. casei* subsp. *pseudo plantarum*, *Sacch. cerevisiae.*
Other details	Kefir 500 mL/day; 4 weeks; no treatment.	Altınkılıc Food and Milk Industry (İstanbul, Turkey): Kefir grains.	100 g kefir: 58 kcal, fat 3.1 g, CHO 4.49 g, protein 9.2 g.	10^10^ cfu *Lc. lactis*/g, 4.7 × 10^3^ cfu *L. pentosus*/g, 7 × 10^7^ cfu *Saccharomyces* spp./g.
Bekar et al. 2011 [56].	Kefir 500 mL/day; 14 days; standard therapy.	Microbiology Laboratory of EKER Corp. (Bursa, Turkey). Details NR.	Details NR.	Details NR.
Cogulu et al. 2010 [57]	Kefir 100–200 mL/day; 3 weeks; milk.	Sakipaga Co. (Izmir, Turkey): kefir culture derived from the Danisco-Biolacta Company’s collection (Olsztyn, Poland).	Milk fat 3%, lactose 3.45%, protein 3.6%.	*Lc. lactis*, *Lc. cremoris*, *Lc. diacetylactis*, *Lu. cremoris*, *L. kefiri*, *Kl. marxianus*, *Sacch. unisporus*, *Streptococcus* spp. 3.3 × 10^8^ cfu *Lactobacillus* spp./mL, 1.7 × 10^9^ cfu *Streptococcus* spp./mL
Can et al. 2009 [58].	Kefir 500 mL/day; CT-dependent; no treatment.	Altinkilic Company (Istanbul, Turkey): kefir grains.	Details NR.	Details NR.
Merenstein et al. 2009 [59].	Kefir 75–150 mL/day; 10 days; inactivated kefir.	Probugs (Lifeway Foods, Inc., Chicago, IL, USA). Details NR.	Details NR.	*Lc. lactis*, *L. plantarum*, *L. rhamnosus*, *L. casei*, *Lc. diacetylactis*, *Lu. cremoris*, *Bifidobacterium longum*, *B. breve*, *L. acidophilus*, *Sacch. florentinus.*
Topuz et al. 2008 [60].	Kefir 500 mL/day; CT-dependent; saline.	Industrial kefir: kefir grains. Details NR.	Details NR.	Details NR.
Hertzler et al. 2003 [61].	Kefir, yogurt (20 g lactose); 1 day each (crossover); milk.	Lifeway Foods (IL, USA): kefir. Details NR. The Dannon Company, Inc., (Tarrytown, New York, NY, USA): yogurt. Kroger Co. (Cincinnati, OH, USA): milk.	20 g lactose: kefir/yogurt/milk—kcal 246–337/252–396/171; fat 4.3–5.25/3.8–5.8/4.2 g; CHO 20.8–44.7/30–73.5/20 g; protein 29/18/13 g. Other details NR.	*St. lactis*, *L. plantarum*, *St. cremoris*, *L. casei*, *St. diacetylactis*, *Sacch. florentinus*, *Lu. cremoris.*
St-Onge et al. 2002 [62].	Kefir 500 mL/day; 12 weeks (crossover); milk.	Liberty Co. Candiac (Quebec, QC, Canada). Details NR.	500 mL kefir/milk: 287 kcal, CHO 31.2 g, fat 7.6 g, protein 23.6 g, cholesterol 31/35 mg.	10^9^ cfu. Details NR.

* Intervention protocol is repeated in abbreviated form to facilitate interpretation of kefir composition and production characteristics. Detailed intervention protocol is noted in Table 2. ** Genera abbreviations: *A.*: *Acetobacter*, *B.*: *Bifidobacterium*. *Ka.*: *Kazachstania*, *Kl.*: *Kluyveromyces*, recently divided lactobacilli [69] were all abbreviated with *L.* including *Lactobacillus* (*acidophilus*, *helveticus*, *lactis*, *kefiranofaciens*, *pentosus*), *Lacticaseibacillus* (*casei*, *rhamnosus*), *Lactiplantibacillus plantarum*, *Lentilactobacillus* (*kefiri* and *parakefiri*), *Lc.*: *Lactococcus*, *Lu*.: *Leuconostoc*, *P.*: *Pichia*, *St.*: *Streptococcus*, *Sacch.*: *Saccharomyces*. Ca: calcium; cfu: colony forming units; CHO: carbohydrates; CT: chemotherapy; Na: sodium; Details NR: details not reported.

## Data Availability

No new data were created.

## References

[1] Turkmen N., Watson R.R., Collier R.J., Preedy V.R. (2017). Chapter 29—Kefir as a Functional Dairy Product. Dairy in Human Health and Disease Across the Lifespan.

[2] Farnworth E.R. (2005). Kefir—A complex probiotic. Food Sci. Technol. Bull. Funct. Foods.

[3] Abou Ayana I.A.A., Al-Otibi F.O., Elgarhy M.R., Omar M.M., El-Abbassy M.Z., Khalifa S.A., Helmy Y.A., Saber W.I.A. (2025). Chemical, Physical, Microbial, and Sensory Properties of Innovative Sesame Milk Kefir, Focusing on the Ultrastructure of Kefir Grains. ACS Omega.

[4] Avila-Reyes S.V., Márquez-Morales C.E., Moreno-León G.R., Jiménez-Aparicio A.R., Arenas-Ocampo M.L., Solorza-Feria J., García-Armenta E., Villalobos-Espinosa J.C. (2022). Comparative Analysis of Fermentation Conditions on the Increase of Biomass and Morphology of Milk Kefir Grains. Appl. Sci..

[5] Yu L., Long M., Zhang G., Lu J., Ding F., Netrusov A., Guo R. (2024). Stimulation of Kefir Grains by Different Juices to Produce Novel Kefirs. Appl. Biochem. Microbiol..

[6] Prado M.R., Blandón L.M., Vandenberghe L.P.S., Rodrigues C., Castro G.R., Thomaz-Soccol V., Soccol C.R. (2015). Milk kefir: Composition, microbial cultures, biological activities, and related products. Front. Microbiol..

[7] Apalowo O.E., Adegoye G.A., Mbogori T., Kandiah J., Obuotor T.M. (2024). Nutritional Characteristics, Health Impact, and Applications of Kefir. Foods.

[8] Garrote G.L., Abraham A.G., De Antoni G.L. (2010). Microbial Interactions in Kefir: A Natural Probiotic Drink. Biotechnology of Lactic Acid Bacteria.

[9] Dahiya D., Nigam P.S. (2023). Therapeutic and Dietary Support for Gastrointestinal Tract Using Kefir as a Nutraceutical Beverage: Dairy-Milk-Based or Plant-Sourced Kefir Probiotic Products for Vegan and Lactose-Intolerant Populations. Fermentation.

[10] Bourrie B.C.T., Willing B.P., Cotter P.D. (2016). The Microbiota and Health Promoting Characteristics of the Fermented Beverage Kefir. Front. Microbiol..

[11] Barukčić I., Gracin L., Jambrak A.R., Božanić R. (2017). Comparison of chemical, rheological and sensory properties of kefir produced by kefir grains and commercial kefir starter. Mljekarstvo.

[12] Otles S., Cagindi O. (2003). Kefir: A Probiotic Dairy-Composition, Nutritional and Therapeutic Aspects. Pak. J. Nutr..

[13] Garmendia G., Gyenes F., Alvarez A., Arbildi E., Giménez S., Gonda M., Vero S. (2025). Dynamic microbiota in water kefir: Microbial shift and ecological selection during fermentation. BMC Microbiol..

[14] Guzel-Seydim Z.B., Gökırmaklı Ç., Greene A.K. (2021). A comparison of milk kefir and water kefir: Physical, chemical, microbiological and functional properties. Trends Food Sci. Technol..

[15] Hill C., Guarner F., Reid G., Gibson G.R., Merenstein D.J., Pot B., Morelli L., Canani R.B., Flint H.J., Salminen S. (2014). The International Scientific Association for Probiotics and Prebiotics consensus statement on the scope and appropriate use of the term probiotic. Nat. Rev. Gastroenterol. Hepatol..

[16] Marco M.L., Sanders M.E., Gänzle M., Arrieta M.C., Cotter P.D., De Vuyst L., Hill C., Holzapfel W., Lebeer S., Merenstein D. (2021). The International Scientific Association for Probiotics and Prebiotics (ISAPP) consensus statement on fermented foods. Nat. Rev. Gastroenterol. Hepatol..

[17] Binda S., Hill C., Johansen E., Obis D., Pot B., Sanders M.E., Tremblay A., Ouwehand A.C. (2020). Criteria to Qualify Microorganisms as “Probiotic” in Foods and Dietary Supplements. Front. Microbiol..

[18] Ghidini M., Nicoletti M., Ratti M., Tomasello G., Lonati V., Ghilardi M., Parati M.C., Borgonovo K., Cabiddu M., Petrelli F. (2021). *Lactobacillus kefiri* LKF01 (Kefibios^®^) for Prevention of Diarrhoea in Cancer Patients Treated with Chemotherapy: A Prospective Study. Nutrients.

[19] Fujioka I., Uchida K. (2025). *Lactococcus cremoris* YRC3780 improves subjective stress response in the Uchida-Kraepelin test: A randomized, double-blind, placebo-controlled study. Sci. Rep..

[20] Toscano M., De Grandi R., Miniello V.L., Mattina R., Drago L. (2017). Ability of Lactobacillus kefiri LKF01 (DSM32079) to colonize the intestinal environment and modify the gut microbiota composition of healthy individuals. Dig. Liver Dis..

[21] Matsuura N., Motoshima H., Uchida K., Yamanaka Y. (2022). Effects of *Lactococcus lactis* subsp. *cremoris* YRC3780 daily intake on the HPA axis response to acute psychological stress in healthy Japanese men. Eur. J. Clin. Nutr..

[22] Wang M.C., Zaydi A.I., Lin W.H., Lin J.S., Liong M.T., Wu J.J. (2020). Putative Probiotic Strains Isolated from Kefir Improve Gastrointestinal Health Parameters in Adults: A Randomized, Single-Blind, Placebo-Controlled Study. Probiotics Antimicrob. Proteins.

[23] Azizi N.F., Kumar M.R., Yeap S.K., Abdullah J.O., Khalid M., Omar A.R., Osman M.A., Mortadza S.A.S., Alitheen N.B. (2021). Kefir and Its Biological Activities. Foods.

[24] Rosa D.D., Dias M.M.S., Grześkowiak Ł.M., Reis S.A., Conceição L.L., Peluzio M.d.C.G. (2017). Milk kefir: Nutritional, microbiological and health benefits. Nutr. Res. Rev..

[25] García-Burgos M., Moreno-Fernández J., Alférez M.J.M., Díaz-Castro J., López-Aliaga I. (2020). New perspectives in fermented dairy products and their health relevance. J. Funct. Foods.

[26] Fekete M., Lehoczki A., Kryczyk-Poprawa A., Zábó V., Varga J.T., Bálint M., Fazekas-Pongor V., Csípő T., Rząsa-Duran E., Varga P. (2025). Functional Foods in Modern Nutrition Science: Mechanisms, Evidence, and Public Health Implications. Nutrients.

[27] Rashidbeygi E., Samarin M.M., Sheikhhossein F., Khalilkhaneh A.H., Gholizadeh M., Lohrasbi N., Abbasi A., Bazyar H., Askari G., Amini M.R. (2025). The Effect of Kefir Consumption on Blood Pressure and C-Reactive Protein: A Systematic Review and Meta-Analysis of Randomised Controlled Trials. Endocrinol. Diabetes Metab..

[28] Hamsho M., Hawari R., Yeşil Z., Dakhel Z., Dursun Saydam D., Terzi M., Ranneh Y. (2025). Effect of different kefir dosages on inflammation status, metabolic profile, and anthropometric measurements in adults: A systematic review and meta-analysis. Nutr. Metab. Cardiovasc. Dis..

[29] Tanure Y.C.B., Mafra A.C.M., Guimarães B.L.M., Magalhães R.C., Fagundez C., Nascimento I., Brito J.C.M. (2025). Potential benefits of kefir and its compounds on Alzheimer’s disease: A systematic review. Brain Behav. Immun. Integr..

[30] Pinto V.R.A., Teixeira C.G., Lima T.S., De Almeida Prata E.R.B., Vidigal M., Martins E., Perrone Í.T., Carvalho A.F. (2020). Health beliefs towards kefir correlate with emotion and attitude: A study using an emoji scale in Brazil. Food Res. Int..

[31] Ganatsios V., Nigam P., Plessas S., Terpou A. (2021). Kefir as a Functional Beverage Gaining Momentum towards Its Health Promoting Attributes. Beverages.

[32] Kairey L., Leech B., El-Assaad F., Bugarcic A., Dawson D., Lauche R. (2023). The effects of kefir consumption on human health: A systematic review of randomized controlled trials. Nutr. Rev..

[33] Salari A., Ghodrat S., Gheflati A., Jarahi L., Hashemi M., Afshari A. (2021). Effect of kefir beverage consumption on glycemic control: A systematic review and meta-analysis of randomized controlled clinical trials. Complement. Ther. Clin. Pract..

[34] Rafie N., Golpour Hamedani S., Ghiasvand R., Miraghajani M. (2015). Kefir and Cancer: A Systematic Review of Literatures. Arch. Iran. Med..

[35] Mohammadi F., Razmjooei N., Mohsenpour M.A., Nejati M.A., Eftekhari M.H., Hejazi N. (2025). The effects of kefir drink on liver aminotransferases and metabolic indicators in patients with nonalcoholic fatty liver disease: A randomized controlled trial. BMC Nutr..

[36] Noori M., Shateri Z., Babajafari S., Eskandari M.H., Parastouei K., Ghasemi M., Afshari H., Samadi M. (2025). The effect of probiotic-fortified kefir on depression, appetite, oxidative stress, and inflammatory parameters in Iranian overweight and obese elderly: A randomized, double-blind, placebo-controlled clinical trial. J. Health Popul. Nutr..

[37] Öneş E., Zavotçu M., Nisan N., Baş M., Sağlam D. (2025). Effects of Kefir Consumption on Gut Microbiota and Athletic Performance in Professional Female Soccer Players: A Randomized Controlled Trial. Nutrients.

[38] Gupta V.K., Rajendraprasad S., Ozkan M., Ramachandran D., Ahmad S., Bakken J.S., Laudanski K., Gajic O., Bauer B., Zec S. (2024). Safety, feasibility, and impact on the gut microbiome of kefir administration in critically ill adults. BMC Med..

[39] Şahin T., Akca G., Özmeriç N. (2024). The role of probiotics for preventing dysbiosis in periodontal disease: A randomized controlled trial. Turk. J. Med. Sci..

[40] Bourrie B.C.T., Forgie A.J., Makarowski A., Cotter P.D., Richard C., Willing B.P. (2023). Consumption of kefir made with traditional microorganisms resulted in greater improvements in LDL cholesterol and plasma markers of inflammation in males when compared to a commercial kefir: A randomized pilot study. Appl. Physiol. Nutr. Metab..

[41] Cannavale C.N., Mysonhimer A.R., Bailey M.A., Cohen N.J., Holscher H.D., Khan N.A. (2023). Consumption of a fermented dairy beverage improves hippocampal-dependent relational memory in a randomized, controlled cross-over trial. Nutr. Neurosci..

[42] Bellikci-Koyu E., Sarer-Yurekli B.P., Karagozlu C., Aydin-Kose F., Ozgen A.G., Buyuktuncer Z. (2022). Probiotic kefir consumption improves serum apolipoprotein A1 levels in metabolic syndrome patients: A randomized controlled clinical trial. Nutr. Res..

[43] Caferoglu Z., Aytekin Sahin G. (2021). The effects of kefir in mixed meals on appetite and food intake: A randomized cross-over trial. Rev. Nutr..

[44] Ghizi A.C.d.S., de Almeida Silva M., Moraes F.S.d.A., da Silva C.L., Endringer D.C., Scherer R., Lenz D., de Lima E.M., Brasil G.A., Maia J.F. (2021). Kefir improves blood parameters and reduces cardiovascular risks in patients with metabolic syndrome. PharmaNutrition.

[45] Pražnikar Z.J., Kenig S., Vardjan T., Bizjak M., Petelin A. (2020). Effects of kefir or milk supplementation on zonulin in overweight subjects. J. Dairy Sci..

[46] Bellikci-Koyu E., Sarer-Yurekli B.P., Akyon Y., Aydin-Kose F., Karagozlu C., Ozgen A.G., Brinkmann A., Nitsche A., Ergunay K., Yilmaz E. (2019). Effects of Regular Kefir Consumption on Gut Microbiota in Patients with Metabolic Syndrome: A Parallel-Group, Randomized, Controlled Study. Nutrients.

[47] Yılmaz İ., Dolar M.E., Özpınar H. (2019). Effect of administering kefir on the changes in fecal microbiota and symptoms of inflammatory bowel disease: A randomized controlled trial. Turk. J. Gastroenterol..

[48] Alp S., Baka Z.M. (2018). Effects of probiotics on salivary *Streptecoccus* mutans and *Lactobacillus* levels in orthodontic patients. Am. J. Orthod. Dentofac. Orthop..

[49] Fathi Y., Ghodrati N., Zibaeenezhad M.J., Faghih S. (2017). Kefir drink causes a significant yet similar improvement in serum lipid profile, compared with low-fat milk, in a dairy-rich diet in overweight or obese premenopausal women: A randomized controlled trial. J. Clin. Lipidol..

[50] Gölünük S.B., Ötztşan N., Sözen H., Koca B. (2017). Effects of traditional fermented beverages on some blood parameters in aerobic exercises. Biomed. Res..

[51] Fathi Y., Faghih S., Zibaeenezhad M.J., Tabatabaei S.H. (2016). Kefir drink leads to a similar weight loss, compared with milk, in a dairy-rich non-energy-restricted diet in overweight or obese premenopausal women: A randomized controlled trial. Eur. J. Nutr..

[52] Kong K.L., Hendrich S. (2012). Glycemic index, insulinemic index, and satiety index of kefir. J. Am. Coll. Nutr..

[53] O’Brien K.V., Stewart L.K., Forney L.A., Aryana K.J., Prinyawiwatkul W., Boeneke C.A. (2015). The effects of postexercise consumption of a kefir beverage on performance and recovery during intensive endurance training. J. Dairy Sci..

[54] Ghasempour M., Sefidgar S.A., Moghadamnia A.A., Ghadimi R., Gharekhani S., Shirkhani L. (2014). Comparative study of Kefir yogurt-drink and sodium fluoride mouth rinse on salivary mutans streptococci. J. Contemp. Dent. Pr..

[55] Turan İ., Dedeli Ö., Bor S., İlter T. (2014). Effects of a kefir supplement on symptoms, colonic transit, and bowel satisfaction score in patients with chronic constipation: A pilot study. Turk. J. Gastroenterol..

[56] Bekar O., Yilmaz Y., Gulten M. (2011). Kefir improves the efficacy and tolerability of triple therapy in eradicating *Helicobacter pylori*. J. Med. Food.

[57] Cogulu D., Topaloglu-Ak A., Caglar E., Sandalli N., Karagozlu C., Ersin N., Yerlikaya O. (2010). Potential effects of a multistrain probiotic-kefir on salivary *Streptococcus mutans* and *Lactobacillus* spp. J. Dent. Sci..

[58] Can G., Topuz E., Derin D., Durna Z., Aydiner A. (2009). Effect of kefir on the quality of life of patients being treated for colorectal cancer. Oncol. Nurs. Forum.

[59] Merenstein D.J., Foster J., D’Amico F. (2009). A randomized clinical trial measuring the influence of kefir on antibiotic-associated diarrhea: The measuring the influence of Kefir (MILK) Study. Arch. Pediatr. Adolesc. Med..

[60] Topuz E., Derin D., Can G., Kürklü E., Cinar S., Aykan F., Cevikbaş A., Dişçi R., Durna Z., Sakar B. (2008). Effect of oral administration of kefir on serum proinflammatory cytokines on 5-FU induced oral mucositis in patients with colorectal cancer. Investig. New Drugs.

[61] Hertzler S.R., Clancy S.M. (2003). Kefir improves lactose digestion and tolerance in adults with lactose maldigestion. J. Am. Diet. Assoc..

[62] St-Onge M.P., Farnworth E.R., Savard T., Chabot D., Mafu A., Jones P.J. (2002). Kefir consumption does not alter plasma lipid levels or cholesterol fractional synthesis rates relative to milk in hyperlipidemic men: A randomized controlled trial [ISRCTN10820810]. BMC Complement. Altern. Med..

[63] Hulley S.B., Cummings S.R., Browner W.S., Grady D.G., Newman T.B. (2013). Designing Clinical Research.

[64] Schulz K.F., Altman D.G., Moher D. (2010). CONSORT 2010 statement: Updated guidelines for reporting parallel group randomised trials. BMJ.

[65] Oren A., Garrity G.M. (2021). Valid publication of the names of forty-two phyla of prokaryotes. Int. J. Syst. Evol. Microbiol..

[66] Tu M.Y., Chen H.L., Tung Y.T., Kao C.C., Hu F.C., Chen C.M. (2015). Short-Term Effects of Kefir-Fermented Milk Consumption on Bone Mineral Density and Bone Metabolism in a Randomized Clinical Trial of Osteoporotic Patients. PLoS ONE.

[67] (2003). Codex Standard for Fermented Milks.

[68] Justel M.A., Outeiriño E.B., Guerra N.P. (2025). Production of Kefir and Kefir-like Beverages: Fundamental Aspects, Advances, and Future Challenges. Processes.

[69] Zheng J., Wittouck S., Salvetti E., Franz C.M.A.P., Harris H.M.B., Mattarelli P., O’Toole P.W., Pot B., Vandamme P., Walter J. (2020). A taxonomic note on the genus *Lactobacillus*: Description of 23 novel genera, emended description of the genus *Lactobacillus* Beijerinck 1901, and union of Lactobacillaceae and Leuconostocaceae. Int. J. Syst. Evol. Microbiol..

[70] Choi Y., Keum G.B., Kang J., Doo H., Kwak J., Kim H., Chae Y., Lee S., Yang H., Kim S. (2025). Evaluation of kefir consumption on gut microbial diversity in a healthy young population using full-length 16S rRNA sequencing. Front. Microbiol..

[71] Vieira C.P., Rosario A., Lelis C.A., Rekowsky B.S.S., Carvalho A.P.A., Rosário D.K.A., Elias T.A., Costa M.P., Foguel D., Conte-Junior C.A. (2021). Bioactive Compounds from Kefir and Their Potential Benefits on Health: A Systematic Review and Meta-Analysis. Oxidative Med. Cell. Longev..

[72] Fitsum S., Gebreyohannes G., Sbhatu D.B. (2025). Bioactive compounds in fermented foods: Health benefits, safety, and future perspectives. Appl. Food Res..

[73] Qaisrani Z.N., Lin W.P., Lay B.B., Phyo K.Y., San M.M., Awaeloh N., Aunsorn S., Pattanayaiying R., Na Ayudthaya S.P., Hongkulsup C. (2025). The Impact of Kefir Consumption on Inflammation, Oxidative Stress Status, and Metabolic-Syndrome-Related Parameters in Animal Models: A Systematic Review and Meta-Analysis. Foods.

[74] Lloyd-Price J., Abu-Ali G., Huttenhower C. (2016). The healthy human microbiome. Genome Med..

[75] Van Hul M., Cani P.D., Petitfils C., De Vos W.M., Tilg H., El-Omar E.M. (2024). What defines a healthy gut microbiome?. Gut.

[76] Human Microbiome Project Consortium (2012). Structure, function and diversity of the healthy human microbiome. Nature.

[77] Marco M.L., Cunningham M., Bischoff S.C., Clarke G., Delzenne N., Lewis J.D., Meisel M., Merenstein D., O’Toole P.W., Staudacher H.M. (2026). The International Scientific Association for Probiotics and Prebiotics (ISAPP) consensus statement on the definition and scope of gut health. Nat. Rev. Gastroenterol. Hepatol..

[78] Nam N.N., Do H.D.K., Loan Trinh K.T., Lee N.Y. (2023). Metagenomics: An Effective Approach for Exploring Microbial Diversity and Functions. Foods.

[79] Go D., Yeon G.-H., Park S.J., Lee Y., Koh H.G., Koo H., Kim K.H., Jin Y.-S., Sung B.H., Kim J. (2024). Integration of metabolomics and other omics: From microbes to microbiome. Appl. Microbiol. Biotechnol..

[80] Zhang X., Li L., Butcher J., Stintzi A., Figeys D. (2019). Advancing functional and translational microbiome research using meta-omics approaches. Microbiome.

[81] Magro D., Venezia M., Rita Balistreri C. (2024). The omics technologies and liquid biopsies: Advantages, limitations, applications. Med. Omics.

[82] Nkuna R., Mohlomi N., Matambo T.S. (2026). From Omics to Applications: How Bioinformatics and Multi-Omics Approaches Are Revolutionizing Metal Bioleaching. Minerals.

[83] Yahyapoor F., Haghighat N., Sohrabi Z., Asbaghi O., Bagherniya M., Jamialahmadi T., Sahebkar A. (2023). Effects of Kefir Consumption on Cardiometabolic Risk Factors: A Systematic Review and Meta-analysis of Randomized Controlled Trials. Curr. Drug Targets.

[84] Bourrie B.C.T., Cotter P.D., Willing B.P. (2018). Traditional kefir reduces weight gain and improves plasma and liver lipid profiles more successfully than a commercial equivalent in a mouse model of obesity. J. Funct. Foods.

[85] Yen C.C., Tsai C.L., Chang G.R., Ko C.H., Tu M.Y., Lan Y.W., Chen H.L., Chen C.M. (2025). Kefir-derived exopolysaccharide ameliorates hyperglycemic control and beta cell integrity in a rat model of type 2 diabetes mellitus. Nutr. Diabetes.

[86] Ghoneum M., Abdulmalek S., Pan D. (2020). Reversal of age-associated oxidative stress in mice by PFT, a novel kefir product. Int. J. Immunopathol. Pharmacol..

[87] Silva A.O., Ribeiro J.M., Patrocínio T.B., Amorim G.E., Pereira-Júnior A.A., Ângelo M.L., de Araújo Paula F.B., de Mello Silva Oliveira N., Ruginsk S.G., Antunes-Rodrigues J. (2023). Protective Effects of Kefir Against Unpredictable Chronic Stress Alterations in Mice Central Nervous System, Heart, and Kidney. Probiotics Antimicrob. Proteins.

[88] Salah N., Eissa S., Mansour A., El Magd N.M.A., Hasanin A.H., El Mahdy M.M., Hassan M.K., Matboli M. (2023). Evaluation of the role of kefir in management of non-alcoholic steatohepatitis rat model via modulation of NASH linked mRNA-miRNA panel. Sci. Rep..

[89] Chen Y.H., Chen H.L., Fan H.C., Tung Y.T., Kuo C.W., Tu M.Y., Chen C.M. (2020). Anti-Inflammatory, Antioxidant, and Antifibrotic Effects of Kefir Peptides on Salt-Induced Renal Vascular Damage and Dysfunction in Aged Stroke-Prone Spontaneously Hypertensive Rats. Antioxidants.

[90] Mert H., Yılmaz H., Irak K., Yıldırım S., Mert N. (2018). Investigation of the Protective Effect of Kefir against Isoproterenol Induced Myocardial Infarction in Rats. Korean J. Food Sci. Anim. Resour..

[91] Albuquerque Pereira M.F., Matias Albuini F., Gouveia Peluzio M.D.C. (2024). Anti-inflammatory pathways of kefir in murine model: A systematic review. Nutr. Rev..

[92] Culpepper T. (2022). The Effects of Kefir and Kefir Components on Immune and Metabolic Physiology in Pre-Clinical Studies: A Narrative Review. Cureus.

[93] Nadelman P., Magno M.B., Masterson D., da Cruz A.G., Maia L.C. (2018). Are dairy products containing probiotics beneficial for oral health? A systematic review and meta-analysis. Clin. Oral Investig..

[94] Farias da Cruz M., Baraúna Magno M., Alves Jural L., Pimentel T.C., Masterson Tavares Pereira Ferreira D., Almeida Esmerino E., Luis Paiva Anciens Ramos G., Vicente Gomila J., Cristina Silva M., Cruz A.G.D. (2022). Probiotics and dairy products in dentistry: A bibliometric and critical review of randomized clinical trials. Food Res. Int..

[95] Sakaryalı Uyar D., Üsküdar Güçlü A., Çelik E., Memiş Özgül B., Altay Koçak A., Başustaoğlu A.C. (2024). Evaluation of probiotics’ efficiency on cariogenic bacteria: Randomized controlled clinical study. BMC Oral Health.

[96] Selvarajan N.B., Vasaviah S.K., Krishnan R. (2020). A Comparative Study to Evaluate the Effects of Probiotic Curd on *Streptococcus mutans*, *Bifidobacterium dentium*, and pH of Saliva in Caries-free Children: An In Vivo Study. J. Pharm. Bioallied Sci..

[97] Black E.G., Bugarcic A., Lauche R., El-Omar E., El-Assaad F. (2025). The Effects of Kefir on the Human Oral and Gut Microbiome. Nutrients.

[98] Walsh L.H., Walsh A.M., Garcia-Perez I., Crispie F., Costabile A., Ellis R., Finlayson J., Finnegan L.A., Claesson M.J., Holmes E. (2023). Comparison of the relative impacts of acute consumption of an inulin-enriched diet, milk kefir or a commercial probiotic product on the human gut microbiome and metabolome. npj Sci. Food.

[99] Peluzio M.D.C.G., Dias M.D.M.E., Martinez J.A., Milagro F.I. (2021). Kefir and Intestinal Microbiota Modulation: Implications in Human Health. Front. Nutr..

[100] Pimentel G., Burton K.J., von Ah U., Bütikofer U., Pralong F.P., Vionnet N., Portmann R., Vergères G. (2018). Metabolic Footprinting of Fermented Milk Consumption in Serum of Healthy Men. J. Nutr..

[101] El-Bashiti T.A., Zabut B.M., Abu Safia F.F. (2019). Effect of Probiotic Fermented Milk (Kefir) on Some Blood Biochemical Parameters Among Newly Diagnosed Type 2 Diabetic Adult Males in Gaza Governorate. Curr. Res. Nutr. Food Sci. J..

[102] Cha J., Kim Y.B., Park S.-E., Lee S.H., Roh S.W., Son H.-S., Whon T.W. (2024). Does kimchi deserve the status of a probiotic food?. Crit. Rev. Food Sci. Nutr..

[103] Figler M., Mózsik G., Schaffer B., Gasztonyi B., Acs P., Szili B., Rab R., Szakály S. (2006). Effect of special Hungarian probiotic kefir on faecal microflora. World J. Gastroenterol..

[104] Ostadrahimi A., Taghizadeh A., Mobasseri M., Farrin N., Payahoo L., Beyramalipoor Gheshlaghi Z., Vahedjabbari M. (2015). Effect of probiotic fermented milk (kefir) on glycemic control and lipid profile in type 2 diabetic patients: A randomized double-blind placebo-controlled clinical trial. Iran. J. Public Health.

[105] Alihosseini N., Moahboob S.A., Farrin N., Mobasseri M., Taghizadeh A., Ostadrahimi A.R. (2017). Effect of Probiotic Fermented Milk (Kefir) on Serum Level of Insulin and Homocysteine in Type 2 Diabetes Patients. Acta Endocrinol..

[106] de Araujo G.V., de Lorena V.M.B., Montenegro S.M.L., de Albuquerque E.C., Peixoto D.M., Sarinho E.S.C. (2017). Probiotics as an adjunct for the treatment of recurrent wheezing in infants and effects on expression of T-helper 1 and regulatory T cytokines. J. Funct. Foods.

[107] Plessas S., Nouska C., Mantzourani I., Kourkoutas Y., Alexopoulos A., Bezirtzoglou E. (2016). Microbiological Exploration of Different Types of Kefir Grains. Fermentation.

[108] Nejati F., Junne S., Neubauer P. (2020). A Big World in Small Grain: A Review of Natural Milk Kefir Starters. Microorganisms.

[109] Fiorda F.A., de Melo Pereira G.V., Thomaz-Soccol V., Rakshit S.K., Pagnoncelli M.G.B., Vandenberghe L.P.d.S., Soccol C.R. (2017). Microbiological, biochemical, and functional aspects of sugary kefir fermentation—A review. Food Microbiol..

[110] Walsh A.M., Crispie F., Kilcawley K., O’Sullivan O., O’Sullivan M.G., Claesson M.J., Cotter P.D. (2016). Microbial Succession and Flavor Production in the Fermented Dairy Beverage Kefir. mSystems.

[111] Zheng Y., Xu L., Zhang S., Liu Y., Ni J., Xiao G. (2023). Effect of a probiotic formula on gastrointestinal health, immune responses and metabolic health in adults with functional constipation or functional diarrhea. Front. Nutr..

[112] Pugh J.N., Sparks A.S., Doran D.A., Fleming S.C., Langan-Evans C., Kirk B., Fearn R., Morton J.P., Close G.L. (2019). Four weeks of probiotic supplementation reduces GI symptoms during a marathon race. Eur. J. Appl. Physiol..

[113] Strauss M., Micetic Turk D., Lorber M., Pogacar M.S., Kozelj A., Tusek Bunc K., Fijan S. (2023). The Multi-Strain Probiotic OMNi-BiOTiC^®^ Active Reduces the Duration of Acute Upper Respiratory Disease in Older People: A Double-Blind, Randomised, Controlled Clinical Trial. Microorganisms.

[114] Łoniewski I., Szulińska M., Kaczmarczyk M., Podsiadło K., Styburski D., Skonieczna-Żydecka K., Bogdański P. (2023). Multispecies probiotic affects fecal short-chain fatty acids in postmenopausal women with obesity: A post hoc analysis of a randomized, double-blind, placebo-controlled study. Nutrition.

[115] Zamani B., Golkar H.R., Farshbaf S., Emadi-Baygi M., Tajabadi-Ebrahimi M., Jafari P., Akhavan R., Taghizadeh M., Memarzadeh M.R., Asemi Z. (2016). Expression of Concern: Clinical and metabolic response to probiotic supplementation in patients with rheumatoid arthritis: A randomized, double-blind, placebo-controlled trial. Int. J. Rheum. Dis..

[116] Marco M.L., Heeney D., Binda S., Cifelli C.J., Cotter P.D., Foligné B., Gänzle M., Kort R., Pasin G., Pihlanto A. (2017). Health benefits of fermented foods: Microbiota and beyond. Curr. Opin. Biotechnol..

[117] Dotsenko V.A., Kononenko I.A. (2012). Dietary correction of nutrition status in patients with irritable bowel syndrome. Vopr. Pitan..

[118] Pilipenko V.I., Burliaeva E.A., Shakhovskaia A.K., Isakov V.A. (2009). Efficacy of using inulin fortified fermented milk products in patients with functional constipation. Vopr. Pitan..

